# Curcumin Regulates Anti-Inflammatory Responses by JAK/STAT/SOCS Signaling Pathway in BV-2 Microglial Cells

**DOI:** 10.3390/biology8030051

**Published:** 2019-06-27

**Authors:** Chiara Porro, Antonia Cianciulli, Teresa Trotta, Dario Domenico Lofrumento, Maria Antonietta Panaro

**Affiliations:** 1 Department of Clinical and Experimental Medicine, University of Foggia, 71100 Foggia, Italy; 2 Department of Biosciences, Biotechnologies and Biopharmaceutics, University of Bari, 70126 Bari, Italy; 3 Department of Biological and Environmental Sciences and Technologies, Section of Human Anatomy, University of Salento, 73100 Lecce, Italy

**Keywords:** microglia, SOCS-1, curcumin, JAK/STAT, IL-4, IL-10

## Abstract

Microglia play important physiological roles in central nervous system (CNS) homeostasis and in the pathogenesis of inflammatory brain diseases. Inflammation stimulates microglia to secrete cytokines and chemokines that guide immune cells to sites of injury/inflammation. Neuroinflammation is also strongly implicated in the pathogenesis of a number of neurodegenerative diseases, including Alzheimer’s disease and Parkinson’s disease, for which nutritional intervention could represent a benefit due to a lack of clinically efficacious drugs. To this end, the anti-inflammatory mechanisms of several phytochemicals, including curcumin, have been extensively studied. The present experiments show that the administration of curcumin is able to increase the production of the anti-inflammatory cytokines, IL-4 and IL-10, in murine BV-2 microglial cells treated with lipopolysaccharide (LPS). Consistent with these data, curcumin stimulation upregulates the expression of Suppressors of cytokine signaling (SOCS)-1, whereas phosphorylation of the JAK2 and STAT3 was reduced. Taken together, these results provide evidence that curcumin is able to regulate neuroinflammatory reactions by eliciting anti-inflammatory responses in microglia through JAK/STAT/SOCS signaling pathway modulation.

## 1. Introduction

Neuroinflammation is involved in the pathogenesis and progression of many neurological disorders, including neurodegenerative diseases. The primary cellular mediators of inflammation in brains affected by neurodegenerative processes are represented by microglia, resident macrophages of the central nervous system (CNS) that are activated by pro-inflammation mediators, aggregated proteins, and bacterially-derived molecules, as well as in response to cell death [1,2]. The innate immune system has developed inflammatory responses in order to limit and prevent damage to and loss of viable cells and tissues. However, if perpetuated, inflammation becomes chronic, with prolonged and intensified expression of pro-inflammatory cytokines and related damaging enzymes, a typical feature of a number of neurodegenerative diseases [3].

Suppressors of cytokine signaling (SOCS) proteins are intracellular proteins able to inhibit cytokine signaling in a wide variety of cell types [4]. They exert their function by binding to phosphorylated tyrosine residues on Janus kinases (JAKs) and/or cytokine receptor subunits, thus disrupting the classical JAK/STAT (signal transducer and activator of transcription) signaling pathway by promoting the proteasomal degradation of activated receptors and removing the stimuli for continued activation.

The two most intensively studied SOCS genes, for their functional aspects, are SOCS-1 and SOCS-3, which play key roles in regulating inflammatory responses. Moreover, among the SOCS protein family, SOCS-1 and SOCS-3 are expressed in microglia, although SOCS-1, but not SOCS-3, can induce the differentiation of M2-like microglial phenotypes [5].

Curcumin is a yellow pigment isolated from the rhizome of the plant *Curcuma longa* (turmeric), widely used as a spice, food additive, and herbal medicine in Asia [6].

In recent years, extensive in vitro and in vivo studies have suggested that curcumin possess anti-inflammatory, antioxidant, and antitumor activities [7,8]. Our previous studies have also shown that curcumin significantly attenuated, in a dose-dependent manner, release of NO and pro-inflammatory cytokines, as well as iNOS expression in LPS-activated microglial cells, preventing PI3K/Akt phosphorylation as well as NF-κB activation [9].

However, the mechanism by which curcumin is able to reduce inflammatory responses of activated microglia is not yet completely understood. Microglial cells play a pivotal role as initiators of inflammatory responses that are strictly regulated by the JAK/STAT/SOCS signaling pathway. In the present study, we explored whether curcumin could be able to modulate microglial responses via the JAK/STAT/SOCS signaling pathway.

The present results may provide critical information that could contribute to the therapeutic use of curcumin in the modulation of microglial activation, and, subsequently, in the prevention of neuroinflammatory diseases.

## 2. Materials and Methods

### 2.1. Cell Cultures and Treatments

The murine BV2 microglia cell line was purchased from the American Type Culture Collection (Manassas, VA, USA). Cells (2 × 10^5^ cells/mL) were cultured in 24 well plates in Dulbecco’s modified Eagle’s medium (DMEM; Invitrogen, Milan, Italy) supplemented with 10% fetal bovine serum (FBS), 100 units/mL penicillin, 100 μg/mL streptomycin, and 2 mM glutamine, (Life Technologies-Invitrogen, Milan, Italy) at 37 °C in a 5% CO_2_ humidified atmosphere for 24 h. The cells were then treated with various concentrations (10, 30, and 50 μM) of curcumin (Sigma-Aldrich, Milan, Italy) for 1 h, and then were stimulated with LPS (1 μg/mL) for 24 h, according to an our previous study [9]. 

### 2.2. Electrophoresis

After the treatments described above, cells were detached, washed by centrifugation at 600 g for 10 min, and lysed by ice-cold lysis buffer [1% (v/v) Triton X-100, 20 mM Tris-HCl, 137 mM NaCl, 10% (v/v) glycerol, 2 mM EDTA, 1mM phenylmethylsulfonyl fluoride (PMSF), 20 µM leupeptin hemisulfate salt, 0.2 U/mL aprotinin (all from Sigma Aldrich)] for 30 min at 4 °C. Lysates were centrifuged at 13,800 g for 20 min at 4 °C, and the supernatants, adjusted to the same protein concentration (20 µg) by Bradford’s protein assay, were subjected to SDS-PAGE (NuPage Electrophoresis System-Invitrogen) in NuPage LDS Sample Buffer 4× 1:4 (v/v) and with NuPage Sample Reducing Agent 1:10 (500 mM dithiothreitol (DTT) at 10× concentration) on 4–12% Novex Bis-Tris Midi gel 1.0 mm precast gels (Life Technologies).

### 2.3. Western Blotting

The proteins resolved by electrophoresis were transferred from the gel to nitrocellulose membranes using iBlot Dry Blotting System A (Life-Technologies). Membranes were then blocked with PBS (pH 7.2), containing 0.1% (v/v) Tween 20 and 5% (w/v) non-fat dried milk for 1 h, and washed three times with 0.1% Tween 20-PBS (T-PBS). Primary antibodies directed against β-actin (1:10000), SOCS-1(1:1000), JAK2 (1:1000), JAK2 (phosphoY1007+Y1008) (1:500), STAT3 (1:1000), STAT3 (phospho Y705) (1:800) (all obtained from Santa Cruz Biotechnology, Inc. (Santa Cruz, Heidelberg, Germany) were incubated overnight. Membranes were incubated with the secondary antibody horseradish peroxidase (HRP)-conjugate (Santa Cruz Biotechnology), diluted 1:2000, for 60 min at room temperature in the dark on a shaker. Immunoreactive bands were visualized by chemiluminescence (BioRad Laboratories, Hercules, CA, USA). The β-actin protein level was used as protein loading control.

The bands obtained after immunoblotting were submitted to densitometric analysis using ID Image Analysis Software (Kodak Digital Science). Results are expressed as arbitrary units.

### 2.4. ELISA

The cytokines released by cultured cells were measured in the supernatants stored at −70 °C. The concentrations of IL-10 and IL-4 were determined by ELISA according to the manufacturer’s protocols (R&D System Inc., Minneapolis, MN, USA).

### 2.5. Reverse Transcriptase–Polymerase Chain Reaction (RT-PCR) and Quantitative Real-Time PCR Analysis

Total RNA was isolated from cells using Trizol Reagent according to the manufacturer’s protocol. The mRNA levels of various genes were quantified using the SYBR Green QuantiTect RTPCR Kit (Roche, South San Francisco, CA, USA). GAPDH was used as endogenous reference.

Data were analyzed using the relative standard curve method according to the manufacturer’s protocol. The mean value of each gene after β-actin normalization at the time point showing the highest expression was used as calibrator to determine the relative levels of cytokine at different time points. The primer sequences for the tested genes are reported in Table 1. 

### 2.6. Treatment of Cell Cultures with Small Interfering RNA

Cell cultures were submitted to SOCS-1 specific small interfering RNA (siRNA) using a commercial kit (Santa Cruz Biotechnology) according to the manufacturer’s protocol. Cells were incubated with 60–80 pmols of control (scrambled) siRNA or SOCS-1 specific siRNA for 5–7 h at 37 °C, using a transfection reagent. Cells were then added to 1 mL of complete medium and incubated for 24 h. The day after, the medium was replaced with fresh culture medium and cells were incubated for 48 h before experimental treatments. Finally, the cells were lysed and SOCS-1 suppression was determined by immunoblotting.

### 2.7. Statistical Analysis

Student’s t test and analysis of variance (one-way ANOVA), followed by Tukey post hoc test, were performed on the results of at least five independent biological replicates. Values of *p* < 0.05 were considered statistically significant.

## 3. Results

### 3.1. Curcumin Regulated Pro-Inflammatory Cytokine Expression in LPS-Treated Cells

**Figure 1:**
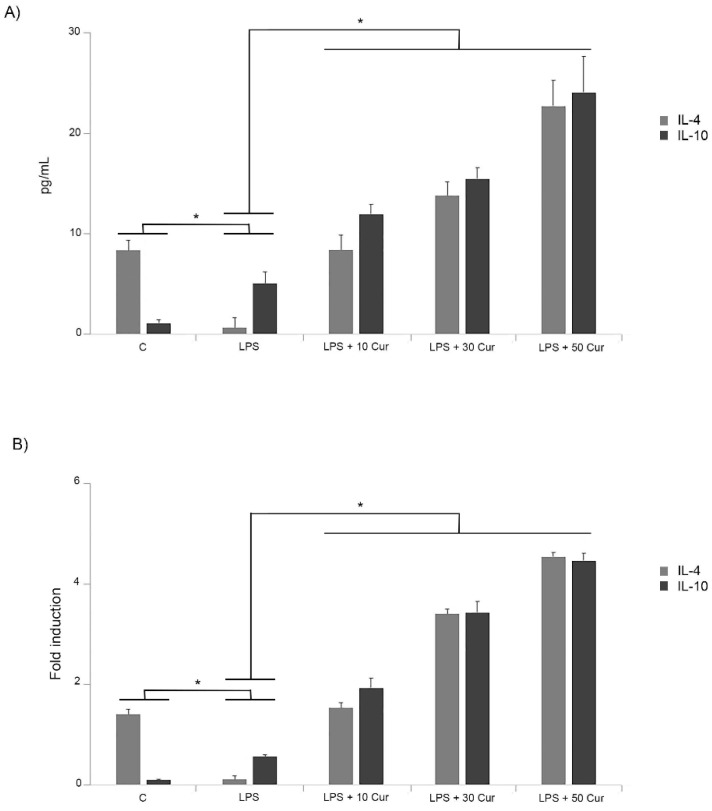
Effect of different concentrations (10, 30, 50 μM) of curcumin (Cur) on anti-inflammatory cytokines production in lipopolysaccharide (LPS)-stimulated BV-2 cells. C = controls. (**A**) ELISA assay. Cells were treated with the indicated concentrations of curcumin for 1 h before LPS (1 μg/mL) treatment. Supernatants were collected at 24 h after LPS treatment, and the amounts of IL-4 and IL-10 were measured. (**B**) Reverse transcriptase–polymerase chain reaction (RT-PCR). BV-2 microglial cells were incubated for 6 h with LPS alone or after 1 h of curcumin pretreatment. The experiments were performed in triplicate, and data are expressed as the mean ± SD of five independent experiments. * *p* < 0.05.

It was reported in our previous paper that the increase in in vitro levels of IL-6, IL-1β, and TNF-α in response to LPS were markedly prevented by treatment with the curcumin at different concentrations [9]. In the present study, we investigated the effect of curcumin on the anti-inflammatory cytokine pattern released by microglia in response to LPS treatment. For this purpose, both ELISA and RT-PCR were performed in order to investigate whether curcumin regulates anti-inflammatory cytokine expression in activated microglia with 1 μg/mL of LPS for 24 h [9]. In a set of experiments, cultured microglia were treated with different concentrations of curcumin. Interestingly, the curcumin treatment significantly increased levels of IL-4 and IL-10 released in supernatants of LPS-activated microglia cells in comparison to LPS-treated cells in the absence of curcumin (Figure 1A).

Although we observed a significant increase of IL-10 in cells stimulated with LPS alone, the administration of curcumin significantly up-regulated IL-10 in a dose-dependent manner. 

Conversely, treatment with LPS inhibited the production of the anti-inflammatory cytokine IL-4, as indicated in Figure 1A. However, treatment of LPS-stimulated cells with curcumin markedly increased IL-4 release in a dose-dependent manner (Figure 1A). These effects of curcumin on the anti-inflammatory cytokine modulation were also confirmed by RT-PCR assay (Figure 1B). Thus, the results indicated that curcumin modulated the expression of these anti-inflammatory cytokines.

### 3.2. Effect of Curcumin on the Signaling Pathway of JAK/STAT/SOCS

**Figure 2:**
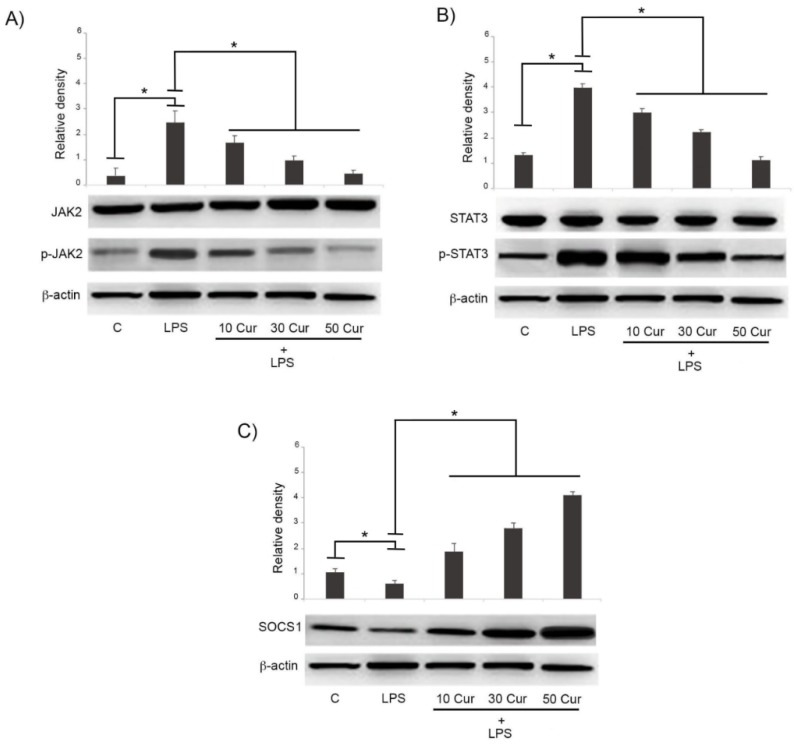
Immunoblot analysis of (**A**) JAK2, (**B**) STAT3, and (**C**) SOCS-1 expression. BV-2 microglial cells were treated for 24 h with 1 μg/mL LPS alone or in the presence of various concentrations (10, 30, 50 μM) of curcumin (Cur). Untreated cells were used as controls (C). Bar graphs represent phospho/total JAK2 (**A**), phospho/total STAT3 (**B**) and SOCS-1 (**C**). Protein expression levels were normalized to β-actin, and results of densitometric analysis are expressed as means ± SD of five independent experiments. * *p* < 0.05.

The JAK/STAT/SOCS signaling pathway actively participates in the cytokine signaling pathway regulation. For this purpose, we investigated by Western blot analysis the effects of different concentrations of curcumin on the expression of these signaling molecules in LPS-activated cells. The expression of p-JAK2 and p-STAT3 were up-regulated in LPS-stimulated cells, indicating the activation of the JAK/STAT signaling pathway. The overexpression of the two biomarkers was significantly reduced in LPS-treated cells exposed to curcumin, indicating that this compound was able to repress, in a dose-dependent manner, the activation of this pathway in microglia, playing a potentially protective effect (Figure 2A,B).

Figure 2 shows that while activation of both JAK2 and STAT3 was significantly reduced, the expression of SOCS-1 was significantly increased in LPS-stimulated cells treated with different concentrations of curcumin. In this case, the modulating effects of curcumin were also shown to be dose-dependent, as reported in Figure 2C.

**Figure 3:**
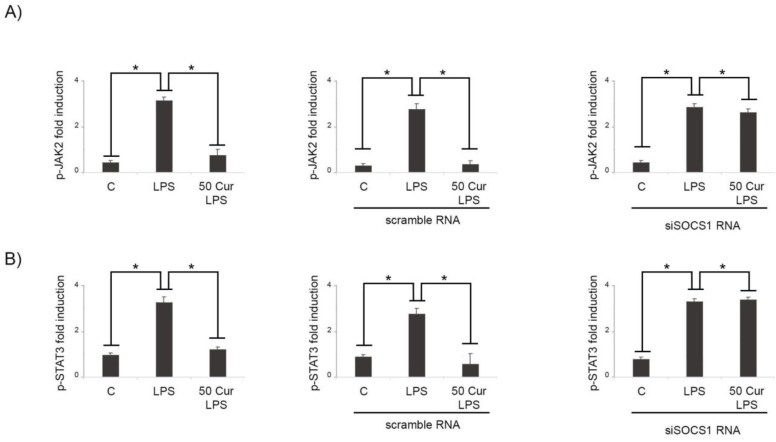
Effects of SOCS-1 silencing on JAK2/STAT3 activation in BV-2 cells. Microglial cells were transfected with either control siRNA (scramble RNA) or SOCS-1 specific siRNA (siSOCS1 RNA). Untransfected cells were used as control (left A and B). (**A**) RT-PCR analysis of p-JAK2 expression in LPS-activated cells (LPS) in absence or in presence of 50 μM curcumin (50 Cur + LPS). (**B**) RT-PCR analysis of p-STAT3 expression in LPS-activated cells (LPS) in absence or in presence of 50 μM curcumin (50 Cur + LPS). Results are expressed as means ± SD of five independent experiments. * *p* < 0.05.

In order to verify a SOCS-1 involvement in the effects induced by curcumin treatment, RNA interfering was used to knock down SOCS-1 expression. Interestingly, siSOCS-1 abrogated the effect of curcumin on JAK2 and STAT3 in LPS-activated cells in comparison to non-silenced microglial cells (Figure 3A), suggesting that curcumin could modulate the JAK/STAT pathway, targeting SOCS-1. In fact, after downregulating SOCS-1 expression in siRNA cells, the mRNA levels of JAK2 and STAT3 both increased significantly. Interestingly, in activated microglia, we observed that in siSOCS-1 cells, curcumin treatment was not able to reduce the expression of p-JAK2 after LPS stimulation (Figure 3A). 

Similarly, we found that in siSOCS-1 microglia cells, the curcumin-induced inhibition of STAT3 activation after LPS treatment was significantly abrogated (Figure 3B). 

### 3.3. Influence of SOCS-1 Downregulation on Microglial Polarization

**Figure 4:**
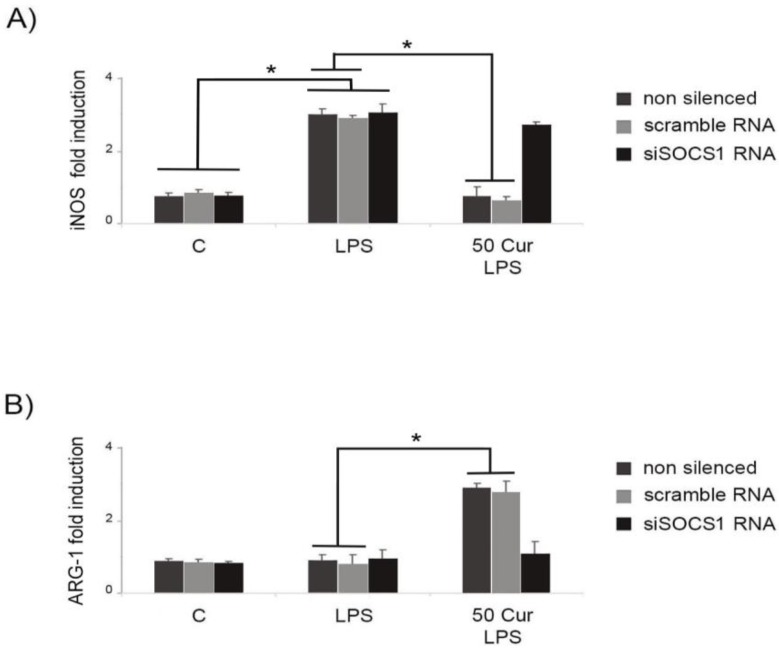
Effect of SOCS-1 downregulation on microglial polarization, determined by RT-PCR. Microglial cells were transfected with either control siRNA (scramble RNA) or SOCS-1 specific siRNA (siSOCS1 RNA). Untransfected cells were used as control. BV-2 microglial cells were incubated for 6 h with LPS alone, or after 1 h of pre-treatment with curcumin (50 μM). (**A**) M1-like (iNOS) expression marker. (**B**) M2-like (ARG-1) expression marker. Experiments were performed in triplicate, and data are expressed as the mean ± SD of five independent experiments. * *p* < 0.05.

iNOS and arginase (ARG)-1 expression were used to discriminate M1-like and M2-like microglial phenotypes, respectively, through surface marker analysis. LPS stimulation induced an evident microglial polarization toward an M1-like-proinflammatory phenotype (Figure 4A). Conversely, curcumin treatment of activated microglia was able to upregulate the expression of ARG-1, a typical M2-like marker (Figure 4B), while at the same time reducing the iNOS expression significantly (Figure 4A,B). 

**Figure 5:**
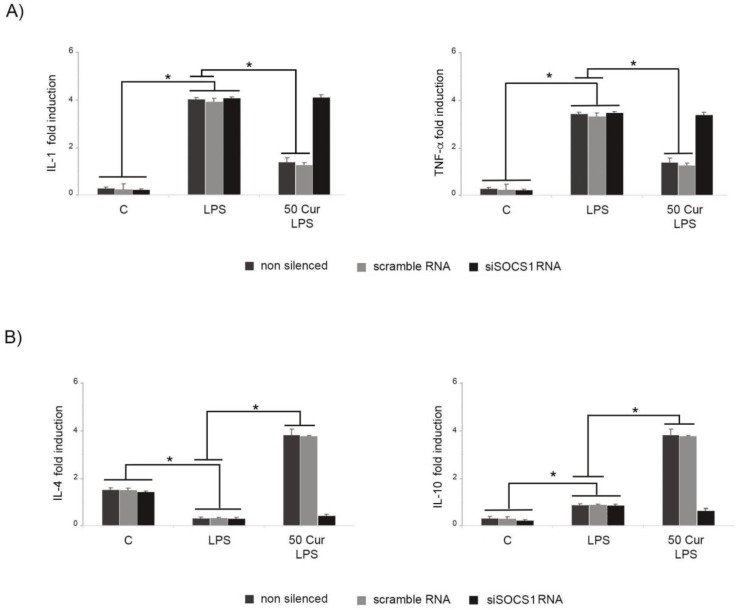
Effect of SOCS-1 downregulation on cytokine production determined by RT-PCR. Microglial cells were transfected with either control siRNA (scramble RNA) or SOCS-1 specific siRNA (siSOCS1 RNA). Untransfected cells were used as control (C). (**A**) Pro-inflammatory cytokine production in LPS-stimulated BV-2 cells. (**B**) Anti-inflammatory cytokine production in LPS-activated BV-2 microglial cells. Cells were incubated for 6 h with LPS alone, or after 1 h of pretreatment with 50 μM curcumin (Cur). Experiments were performed in triplicate, and data are expressed as the mean ± SD of five independent experiments. * *p* < 0.05.

Following down-regulation of SOCS-1 expression, we observed that in silenced cells, the proportion of M1-like cells was significantly increased, while M2-like type cells were significantly diminished, indicating that in siSOCS-1 cells, curcumin treatment is not able to induce a polarization of LPS-activated microglia toward an M2-like phenotype. These results were confirmed by analysis of inflammatory cytokine patterns, evidencing a significant downregulation of the anti-inflammatory responses in siSOCS-1 cells submitted to LPS stimulation in presence of curcumin. In fact, Figure 5 shows that in LPS-activated cells, SOCS-1 down-regulation leads to a significant reduction of anti-inflammatory cytokines, IL-10 and IL-4, also after curcumin treatment, similarly to results observed in activated cells in the absence of curcumin, thus suggesting that curcumin action on functional responses by the M2-like microglia phenotype is linked to SOCS-1 expression.

Contextually, in siSOCS-1 cells, curcumin treatment was unable to downregulate the release of pro-inflammatory cytokines after LPS activation, confirming that SOCS-1 may represent a possible molecular target by which curcumin exerts its regulatory effect on microglial polarization. 

## 4. Discussion

In this study, we evaluated the effects of curcumin in LPS-activated microglial cells. We clearly observed that the activity of the JAK2/STAT3 signal transduction pathway is rapidly increased in microglial cells in response to LPS stimulation, but was significantly diminished after curcumin treatment. In addition, we have shown that curcumin up-regulated SOCS-1 inhibitory protein which efficiently blocked JAK/STAT3 function. Moreover, our results illustrate that curcumin treatment was able to efficiently reduce M1-like/M2-like ratio. The anti-inflammatory effect of curcumin effectively increased the expression level of both IL-10 and IL-4 in LPS-activated microglia. Thus, our results validate the hypothesis that the inhibition of inflammatory activity was mediated by SOCS-1 as well as suppressing the phosphorylation of JAK2/STAT-3.

SOCS-1 silencing abolished curcumin anti-inflammatory effects. SOCS-1-silenced microglia produced enhanced levels of IL-1β and TNF-α in LPS-stimulated cells, and also in the presence of curcumin. Contextually, in siSOCS-1 microglia, we also detected low levels of anti-inflammatory cytokines and a reduction of the M2-like population, shifting microglial responses toward M1-like phenotype. 

The above results indicated that when SOCS-1 expression is downregulated, the JAK2/STAT3 pathway is activated, promoting the polarization of macrophages into M1-like type.

In general terms, M2-like macrophages are activated by Th2 cytokines and can release high amounts of anti-inflammatory cytokines including IL-10 and IL-4, thereby inhibiting the inflammatory response.

It has also been reported by other studies that SOCS-1 is able to inhibit JAK2 phosphorylation through the central SH-2 region and inhibit activation of the STAT3 signaling pathway, thus regulating the secretion of inflammatory cytokines by macrophages [10,11]. Functional loss of SOCS-1 may be responsible for the JAK2/STAT3 activation and pro-inflammatory cytokine accumulation. In contrast, the increased expression of SOCS-1 would inhibit JAK2/STAT3 activation, thus reducing secretion of pro-inflammatory cytokines [12,13]. Therefore, SOCS-1 downregulation may be crucial for JAK/STAT pathway activation, and thereby promote polarization of M1-like macrophages.

These observations suggest that changes in SOCS-1 expression also represent an important regulatory pathway for microglial responses.

Previous observations reported that curcumin did not induce the transcription of SOCS-1 and SOCS-3 in rat primary microglia and BV2 cells [14]; however, in these experiments, different experimental conditions were employed, being microglial cells activated in different experimental conditions, represented by IFN-γ, in the presence of a low dose of curcumin [14]. 

The peculiar function of SOCS is inhibition of signaling by the IL-6 family of cytokines [15]. Interestingly, Zhang et al. [16] found that in rat models of intestinal inflammation, curcumin attenuated inflammatory damages induced by 2,4,6-trinitrobenzene sulfonic acid by enhancing SOCS-1 expression and inhibiting JAK/STATpathways.

SOCS-1 is crucial for limiting the M1-like phenotype in response to IFN-γ/LPS stimulation. It was reported that macrophages from mice deficient in SOCS-1 (and IFN-γ) were hypersensitive to LPS signaling, as shown by enhanced IκB and p38 phosphorylation [17]. SOCS-1 deficiency in these macrophages led to enhanced M1-like gene expression (TNF-α, IL-1β, and IL-6) after LPS stimulation [17]. These observations emphasize the role of SOCS-1 as a negative regulator of TLR-4 signaling.

In our experiments, we observed that increased expression of SOCS-1 after curcumin administration reduced the expression of STAT3 and decreased the production of IL-1β and TNF-α, inducing a M2-like cell response. Similar effects exerted by curcumin, although in another in vitro model, have been described by Li et al. [18].

The anti-inflammatory effect of curcumin has been described in various tissues [19], and a number of researchers have reported that curcumin could exert its protective effects by inhibiting the activation of the JAK/STAT signaling pathway in renal endothelial dysfunction [20], in experimental colitis [21], glioma [22], or in myocardial ischemia reperfusion [23]. However, our study demonstrated for the first time, to our knowledge, that in activated microglia, curcumin is able to up-regulate anti-inflammatory responses, evaluated in terms of IL-10 and IL-4 release, through JAK/STAT/SOCS signaling pathway modulation.

Additionally, we detected levels of cytokines in LPS-activated microglia that are involved in the inflammatory processes, including TNF-α, IL-1β, IL-10, and IL-4. LPS stimulation strongly induced the increase of pro-inflammatory factors TNF-α, IL-1β and a moderate increase of IL-10; however, there was a significant reduction of TNF-α and IL-1β after curcumin treatment, together with a significant increase of the anti-inflammatory cytokines IL-10 and IL-4. 

IL-4 serves as an immunosuppressive cytokine that may inhibit the synthesis of several pro-inflammatory cytokines, as previously reported [24]. In this regard, the anti-inflammatory cytokine IL-4 has been linked to microglia-mediated neuroprotection and neurogenesis [25]. In this paper, we observed that LPS stimulation appears to inhibit IL-4 release, while curcumin treatment enhanced IL-4 production. 

IL-10 controls the inflammatory processes by suppressing the expression of pro-inflammatory cytokines, including TNF-α and IL-6 [26,27].

It is well demonstrated that IL-10 is produced by LPS-stimulated BV-2 cells [28,29]. Our results demonstrated that, although LPS was able to induce a significant release of IL-10, curcumin treatment effectively up-regulated IL-10 by endotoxin-activated BV-2 cells. 

Moreover, in this paper, we observed that LPS stimulation appears to inhibit IL-4 release, while curcumin treatment enhanced IL-4 production. In this context, it was reported that the anti-inflammatory cytokine IL-4 is linked to microglia-mediated neuroprotection and neurogenesis [30,31]. In line with this concept, we observed that LPS stimulation appears to inhibit IL-4 release by BV-2 cells, similarly to previous observations reported by other authors [32]. In addition, it was reported that IL-4 concentration was significantly decreased in microglia exposed to LPS for 24 h, compared with untreated cells, and IL-4 level was considerably decreased in the LPS-treated rat brain [33,34]. Regarding signaling mechanisms probably responsible for IL-4 down-regulation by LPS, it was reported that LPS, acting through TLR4 engagement, mediates both type 1 and type 2 cytokine responses through distinct kinetic patterns both in vitro and in vivo. In particular, rapid MyD88-dependent and TRAM-independent production of TNF-α, and a delayed MyD88 and TRAM–dependent IL-4 production in macrophages have been proposed in order to justify the different cytokine patterns after LPS stimulation [35]. 

Interestingly, we observed that curcumin treatment enhanced not only IL-10, but also IL-4 production, thus highlighting that regulation of the expression of anti-inflammatory cytokines may represent an important factor in the modulation of microglial responses by curcumin during neuroinflammation.

Moreover, analyzing the M1-like/M2-like ratio, we clearly observed that curcumin treatment acted on microglia polarization, favoring a shifting toward the M2-like phenotype, thus implying a beneficial effect of curcumin for anti-inflammatory activity.

The role of SOCS proteins in macrophage polarization was previously reported [36,37]. These authors observed, in SOCS-2 and SOCS-3 knockout mice, that these proteins are important regulatory molecules for polarization into M1-like and M2-like macrophages and inflammatory responses. The SOCS-2 knockout macrophages highly expressed M1-like markers, while SOCS-3 knockout macrophages highly expressed M2-like markers, indicating that SOCS-2 plays an important role in polarization into M2-like macrophages and inhibition of inflammatory responses. In this regard, SOCS-3 is involved in macrophage polarization into M1-like, thus promoting the occurrence of inflammatory responses [38]. The present study demonstrates that curcumin-induced SOCS-1 exerts a key role in the regulation of microglial polarization, evidenced by IL-4 and IL-10 release, markers typical of M2 responses.

There is similar evidence that manipulation of SOCS expression, particularly SOCS-1, could represent a target for drug development aimed to counteract inflammation. Cai et al. [39] reported that inflammatory responses in Aβ− treated N9 microglial cells were reduced through the SOCS-1 up-regulation, enhancing the levels of M2-like markers, including ARG-1 and IL-10, and that these protective effects were abolished by SOCS-1 siRNA. 

In vivo, the role of inflammation in both pathogenesis and progression of neurodegenerative disease has been demonstrated in many model studies, in which the benefits of phytochemicals in the reduction of the neuroinflammatory processes have been widely investigated. In particular, it has been shown that curcumin tends to reduce the expression of pro-inflammatory mediators, such as IL-6 and TNF-α, as well as ROS in PD and AD animal models [40]. 

Our results indicated that inactivation of the JAK/STAT signal limited the extent of the secretion of pro-inflammatory cytokines, leading to an increase in the level of IL-4 and IL-10. This process leads to a shift of microglial polarization toward a protective phenotype, thus alleviating inflammatory processes that exacerbate neurodegeneration.

These results suggest that the beneficial effects of curcumin observed in vivo could also be exerted by the enhancement of anti-inflammatory responses, as well as the already characterized inhibition of pro-inflammatory responses.

## 5. Conclusions

In this study, we found that curcumin inhibited phosphorylation of JAK2 and STAT3 in LPS-activated microglia and increased expression of SOCS-1 suppressing the activation of the JAK/STAT signal. These effects demonstrated for the first time that curcumin proved to be not only a potent inhibitor of pro-inflammatory responses, but also a valid modulator of cytokine production by activated microglia. These findings underpin the immunopharmacological roles of curcumin as a potentially effective drug for the control of neuroinflammatory diseases.

## Figures and Tables

**Table 1:** DNA sequences of primers used in PCR reactions.

Gene	Sequence 5’→ 3’	Sequence Reference
IL-4	Fw-ATCATCGGCATTTTGAACGAGGTC Rw-ACCTTGGAAGCCCTACAGACGA	NM_021283.2
IL-10	Fw-GCCAGTACAGCCGGGAAGACAATA Rw- GCCTTGTAGACACCTTGGTCTT	NM_012854.2
TNF-α	Fw- GGCAGGTCTACTTTGGAGTCATTGC Rw- ACATTCGAGGCTCCAGTGAATTCGG	NM_013693.2
IL-1β	Fw- GCAGCAGCACATCAACAAGAGC Rw- TGTCCTCATCCTGGAAGGTCCACG	NM_008361.2
SOCS-1	Fw- TGGGCACCTTCTTGGTGCGC Rw- GGCAGTCGAAGGTCTCGCGG	BC_132368.1
iNOS	Fw- CAACAGGGAGAAAGCGCAAA Rw- TGATGGACCCCAAGCAAGAC	NM_001313921.1
Arg-1	Fw- TTTCAGGACTAGATATCATGGAAGTG Rw- CTTAGGTGGTTTAAGGTAGTCAGTCC	U_51805.1
β-actin	Fw- GACCTCTATGCCAACACAGT Rw- AGTACTTGCGCTCAGGAGGA	NM_007393.5
